# A database for ITS2 sequences from nematodes

**DOI:** 10.1186/s12863-020-00880-0

**Published:** 2020-07-10

**Authors:** Matthew L. Workentine, Rebecca Chen, Shawna Zhu, Stefan Gavriliuc, Nicolette Shaw, Jill de Rijke, Elizabeth M. Redman, Russell W. Avramenko, Janneke Wit, Jocelyn Poissant, John S. Gilleard

**Affiliations:** 1 Department of Comparative Biology and Experimental Medicine, Faculty of Veterinary Medicine, Calgary, Canada; 2 Ecosystem and Public Health, Faculty of Veterinary Medicine, Calgary, Canada

**Keywords:** Database, ITS2, Amplicon, Sequencing

## Abstract

**Background:**

Marker gene surveys have a wide variety of applications in species identification, population genetics, and molecular epidemiology. As these methods expand to new types of organisms and additional markers beyond 16S and 18S rRNA genes, comprehensive databases are a critical requirement for proper analysis of these data.

**Results:**

Here we present an ITS2 rDNA database for marker gene surveys of both free-living and parasitic nematode populations and the software used to build the database. This is currently the most complete and up-to-date ITS2 database for nematodes and is able to reproduce previous analysis that used a smaller database.

**Conclusions:**

This database is an important resource for researchers working on nematodes and also provides a tool to create ITS2 databases for any given taxonomy.

## Background

The internal transcribed spacer 2 (ITS2) rDNA locus has been widely used as a marker for species identification in both free-living and parasitic nematodes for many years [1–3]. Nematodes, as with other invertebrate groups, often exist in large and complex communities. Consequently, deep amplicon sequencing approaches have a potentially powerful role for the investigation of nematode communities similar to the use of bacterial 16S rDNA amplicon sequencing in microbiome studies. For example, the ITS2 rDNA locus has recently been used for “nemabiome” sequencing of parasitic nematode communities inhabiting the gastrointestinal tract of cattle [4, 5]. In that case, reliable species identification was achieved using a small bespoke, curated ITS2 rDNA database of the major relevant cattle gastrointestinal nematode species. However, the wider and more versatile application of deep amplicon sequencing approaches to nematode research will require a more comprehensive, and regularly updated, ITS2 rDNA database equivalent to that available for studying fungi [6]. A eukaryotic ITS2 database has been previously published [7] but has not been updated since 2015 and contains only 1347 sequences in the Nematoda phylum. To our knowledge no other good ITS2 databases exist for nematodes. In this paper, we describe the development of a nematode ITS2 rDNA database and the software to create ITS2 databases for any taxonomy.

## Implementation

The nematode ITS2 database was constructed using markerDB, which we have provided as an open-source tool to quickly and reliably construct an ITS2 database for any NCBI taxonomic level. This tool is made available to facilitate reproducibility and transparency, and to provide users with the option to construct their own databases. markerDB is implemented in the R programming language [8] and run as a Snakemake [9] pipeline. The software will run on Linux or MacOS and is dependency free with the use of Bioconda [10]. A brief description of the pipeline follows.

Potential ITS2 sequences are retrieved from NCBI using the rentrez R package [11] based on a text search that will find ITS2 annotated sequences that are limited to the provided taxonomy. The full taxonomy of the downloaded sequences is obtained with the taxize R package [12, 13]. Only taxonomies that are complete with all ranks (Superkingdom, Kingdom, Phylum, Class, Order, Family, Genus, Species) are retained. Additionally, taxonomies with incomplete species names, which contain numbers or ‘sp.’ are removed.

Many of the ITS2 annotated sequences also contain the partial or full upstream and downstream 5.8S and 28S genes and so trimming to the ITS2 region is required. However, no good sequence models exist that capture a wide range of diversity due to the divergence of this region. For this step Infernal, (specifically cmscan) [14] is used to identify the 5.8S and eukaryotic LSU (large subunit, 28S) genes. The co-variance models used to create the nematode database and also provided with markerDB, were taken from Rfam [15]. If a hit to the 5.8S is identified (partial hits allowed) this region and everything upstream is trimmed off. This is repeated downstream for any 28S hits. Any retrieved sequences that do not have hits to either rRNA gene are assumed to be solely ITS2 and are retained in the database. This option can be changed when running the pipeline. A similar strategy has been previously used to identify ITS2 sequences [16] however, that approach relied on custom HMM models to locate the ITS sequences which make it challenging keep the database up-to-date. Using publicly available and frequently updated sequence models from Rfam ensures the most current data is used.

Finally, sequences too long or too short (700 bp and 100 bp, respectively, as set in the configuration) are discarded. The final sequence set contains a fair bit of redundancy and so a non-redundant version of the database with unique sequences only is returned. If an alignment is required an option to align the sequences using MAFFT [17] is also provided but it should be noted that aligning ITS2 sequences from diverse organisms is difficult due to the heterogeneity present. In general we recommend taxonomy assignment methods that do not depend on alignments, particularly for databases covering a large taxonomic range.

The output of markerDB is a fasta file with the final sequences and a corresponding tab-delimited text file with the taxonomy, linked by Genbank accession number. The pipeline also provides function to write out the database in formats used with popular taxonomy assignment methods including dada2 [18], the rRDP Bioconductor package [19], mothur [20], and IDTAXA [21]. For example the IDTAXA output files can be used with our recommended nemabiome analysis workflow (www.nemabiome.ca). A simple shiny app is also provided that allows users to work with the database interactively, filtering taxonomic groups as needed and downloading the filtered data in any of the above formats.

## Results and discussion

**Fig. 1:**
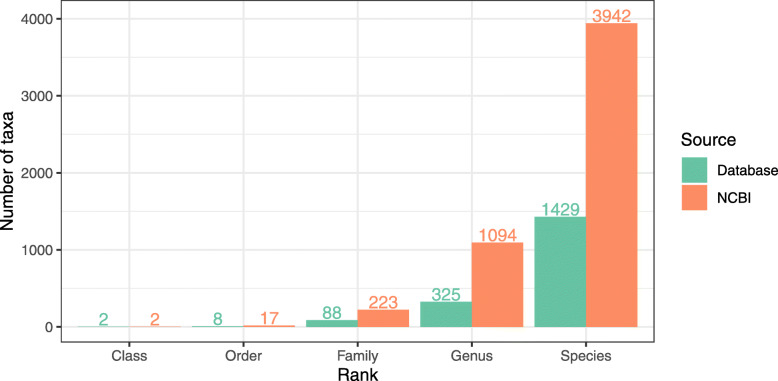
Database size. Number of taxa in version 1 of the ITS2 database compared to the total in the NCBI taxonomy database

The database (version 1.0.0 at the time of writing) currently contains 8630 non-redundant sequences with a median length of 263 bp and standard deviation of 97 bp. There are 1429 species and 325 genera and across the taxonomic ranks we were able to obtain good quality, non-redundant sequences for approximately 30% of the taxa in the NCBI database in the Nematoda phylum (see Fig. 1). We have also included an example of data as an illustration of the use of the ITS-2 rDNA database (Additional file 1: Figure 1).

We have also provided a simple web app, which allows users to search and filter the database and create versions customized to their research area of interest. Further, the database will be updated every 3-6 months, feasible due the automation and reproducibility of the database construction using markerDB. Rapid updates allow researchers to generate analysis that reflect the most current sequences in Genbank.

## Conclusions

In conclusion, we provide a database of nematode ITS2 sequences that greatly expands the range of sequences suitable to study both parasitic and free-living nematode communities allowing a broader selection of hosts and environments to be studied. We have also provided open source software to easily and reproduceably build ITS2 databases for any taxonomy of interest.

## Availability and requirements

- **Project name:** markerDB
- **Project home page:**https://github.com/ucvm/markerDB
- **Operating system:** Unix-based, i.e. MacOS or Linux
- **Programming languange**: R and Python
- **Other requirements:** Bioconda
- **License:** MIT
- **Any resitriction ot use by non-academics:** None

## Supplementary information

**Additional file 1** Supplementary figure 1.

## Data Availability

The database is available as an interactive web app at https://www.nemabiome.ca/its2-database.html. The full database can also be downloaded from zenodo 10.5281/zenodo.3235802, and the open source software used to create the database, markerDB, is freely available at https://github.com/ucvm/markerDB/releases/latest.

## Abbreviations

ITS2: Internal transcribed spacer 2
rDNA: ribosomal DNA

## References

[1] Gasser RB, Monti JR (1997). Identification of parasitic nematodes by PCR-SSCP of ITS-2 rDNA. Mol Cell Probes.

[2] Newton LA, Chilton NB, Beveridge I, Hoste H, Nansen P, Gasser RB (1998). Genetic markers for strongylid nematodes of livestock defined by PCR-based restriction analysis of spacer rDNA. Acta Trop.

[3] Powers TO, Todd TC, Burnell AM, Murray PCB, Fleming CC, Szalanski AL, Adams BA, Harris TS (1997). The rDNA Internal Transcribed Spacer Region as a Taxonomic Marker for Nematodes. J Nematol.

[4] Avramenko RW, Redman EM, Lewis R, Yazwinski TA, Wasmuth JD, Gilleard JS (2015). Exploring the Gastrointestinal "Nemabiome": Deep Amplicon Sequencing to Quantify the Species Composition of Parasitic Nematode Communities. PLoS ONE.

[5] Avramenko RW, Redman EM, Lewis R, Bichuette MA, Palmeira BM, Yazwinski TA, Gilleard JS (2017). The use of nemabiome metabarcoding to explore gastro-intestinal nematode species diversity and anthelmintic treatment effectiveness in beef calves. Int J Parasitol.

[6] Nilsson RH, Larsson K-H, Taylor AFS, Bengtsson-Palme J, Jeppesen TS, Schigel D, Kennedy P, Picard K, Glöckner FO, Tedersoo L, Saar I, Kõljalg U, Abarenkov K (2019). The UNITE database for molecular identification of fungi: Handling dark taxa and parallel taxonomic classifications. Nucleic Acids Res.

[7] Ankenbrand MJ, Keller A, Wolf M, Schultz J, Förster F (2015). ITS2 Database V: Twice as Much. Mol Biol Evol.

[8] R Core Team (2019). R: A Language and Environment for Statistical Computing.

[9] Köster J, Rahmann S. Snakemake—a scalable bioinformatics workflow engine; 28(19):2520–2. 10.1093/bioinformatics/bts480. 10.1093/bioinformatics/bts480 22908215

[10] Grüning B, Dale R, Sjödin A, Chapman BA, Rowe J, Tomkins-Tinch CH, Valieris R, Köster J. Bioconda: Sustainable and comprehensive software distribution for the life sciences. Nat Methods. 2018; 15(7):475–6. 10.1038/s41592-018-0046-7. 10.1038/s41592-018-0046-7 PMC11070151 29967506

[11] Winter DJ (2017). Rentrez: An R package for the NCBI eUtils API. R J.

[12] Chamberlain SA, Szoecs E (2013). Taxize: taxonomic search and retrieval in R [version 2; peer review: 3 approved]. F1000Research.

[13] Chamberlain S, Szöcs E (2013). Taxize - taxonomic search and retrieval in R. F1000Research.

[14] Nawrocki EP, Eddy SR (2013). Infernal 1.1: 100-fold faster RNA homology searches. Bioinformatics.

[15] Kalvari I, Argasinska J, Quinones-Olvera N, Nawrocki EP, Rivas E, Eddy SR, Bateman A, Finn RD, Petrov AI (2018). Rfam 130: Shifting to a genome-centric resource for non-coding RNA families. Nucleic Acids Res.

[16] Bengtsson-Palme J, Ryberg M, Hartmann M, Branco S, Wang Z, Godhe A, Wit PD, Sánchez-García M, Ebersberger I, de Sousa F, Amend A, Jumpponen A, Unterseher M, Kristiansson E, Abarenkov K, Bertrand YJK, Sanli K, Eriksson KM, Vik U, Veldre V, Nilsson RH (2013). Improved software detection and extraction of ITS1 and ITS2 from ribosomal ITS sequences of fungi and other eukaryotes for analysis of environmental sequencing data. Methods Ecol Evol.

[17] Katoh K, Misawa K, Kuma K-i, Miyata T (2002). MAFFT: A novel method for rapid multiple sequence alignment based on fast Fourier transform. Nucleic Acids Res.

[18] Callahan BJ, McMurdie PJ, Rosen MJ, Han AW, Johnson AJA, Holmes SP (2016). DADA2: High-resolution sample inference from Illumina amplicon data. Nat Methods.

[19] Hahsler M, Nagar A. rRDP: Interface to the RDP Classifier. R package version 1.22.0; 2020.

[20] Schloss PD, Westcott SL, Ryabin T, Hall JR, Hartmann M, Hollister EB, Lesniewski RA, Oakley BB, Parks DH, Robinson CJ, Sahl JW, Stres B, Thallinger GG, Van Horn DJ, Weber CF. Introducing mothur: Open-source, platform-independent, community-supported software for describing and comparing microbial communities. Appl Environ Microbiol. 2009; 75(23):7537–41. 10.1128/AEM.01541-09 PMC2786419 19801464

[21] Murali A, Bhargava A, Wright ES (2018). IDTAXA: A novel approach for accurate taxonomic classification of microbiome sequences. Microbiome.

