# Supplementary material for: A database for ITS2 sequences from nematodes
Source: BMC Genet. 2020 Jul 10;21:74. doi: 10.1186/s12863-020-00880-0 (PMC7350610; doi:10.1186/s12863-020-00880-0)

Supplementary Figure 1 Relative species abundance of gastrointsestinal nematode parasites of 32 flocks of ewes across the UK in 2008 estimated using the Nemabiome ITS-2 assay. Sequence reads passed through a Mothur bioinformatic pipeline and classified using the knn method against the newly created ITS-2 database. The number of reads taxonomically assigned per sample ranged from 15,893 to 84,415 (average = 57,055 reads). Classification and relative species abundance of these samples previously estimated using a much smaller manually created database (Redman *et al.,* 2019)

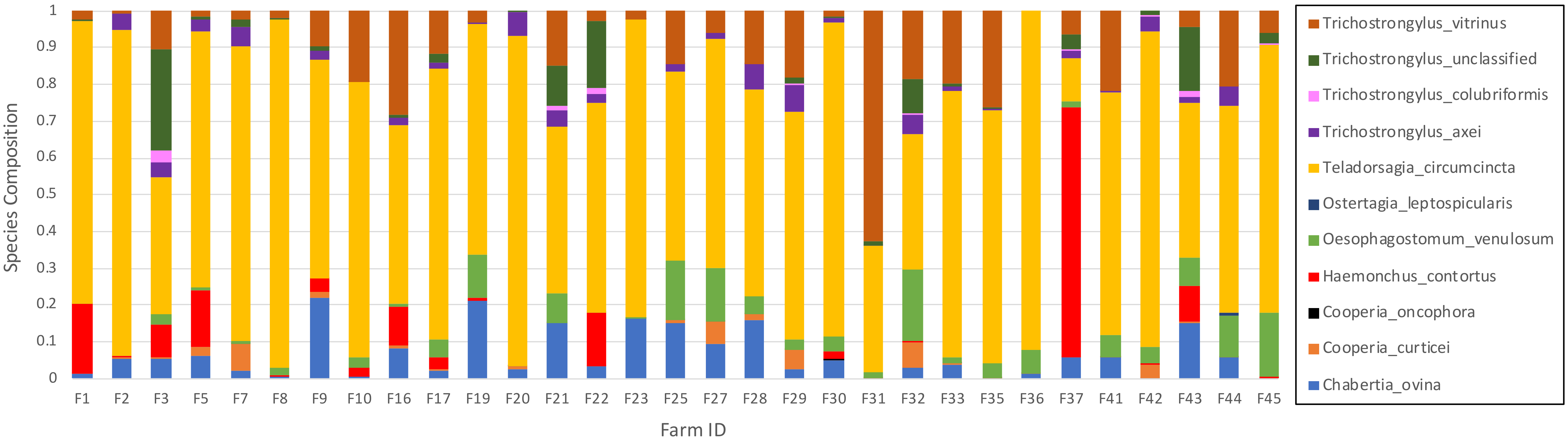

Supplement: Supplementary file 1 — Additional file 1 Supplementary figure 1. [file 12863_2020_880_MOESM1_ESM.pdf]
